# Total 3D Airo® Navigation for Minimally Invasive Transforaminal Lumbar Interbody Fusion

**DOI:** 10.1155/2016/5027340

**Published:** 2016-07-27

**Authors:** Xiaofeng Lian, Rodrigo Navarro-Ramirez, Connor Berlin, Ajit Jada, Yu Moriguchi, Qiwei Zhang, Roger Härtl

**Affiliations:** ^1^Weill Cornell Brain and Spine Center, Department of Neurological Surgery, Weill Cornell Medical College, New York-Presbyterian Hospital, 525 East 68th Street, P.O. Box 99, New York City, NY 10065, USA; ^2^Spine Subdivision, Department of Orthopedics, Shanghai Jiao Tong University Affiliated Sixth People's Hospital, Shanghai, China

## Abstract

*Introduction.* A new generation of iCT scanner, Airo®, has been introduced. The purpose of this study is to describe how Airo facilitates minimally invasive transforaminal lumbar interbody fusion (MIS-TLIF).* Method*. We used the latest generation of portable iCT in all cases without the assistance of K-wires. We recorded the operation time, number of scans, and pedicle screw accuracy.* Results*. From January 2015 to December 2015, 33 consecutive patients consisting of 17 men and 16 women underwent single-level or two-level MIS-TLIF operations in our institution. The ages ranged from 23 years to 86 years (mean, 66.6 years). We treated all the cases in MIS fashion. In four cases, a tubular laminectomy at L1/2 was performed at the same time. The average operation time was 192.8 minutes and average time of placement per screw was 2.6 minutes. No additional fluoroscopy was used. Our screw accuracy rate was 98.6%. No complications were encountered.* Conclusions*. Airo iCT MIS-TLIF can be used for initial planning of the skin incision, precise screw, and cage placement, without the need for fluoroscopy. “Total navigation” (complete intraoperative 3D navigation without fluoroscopy) can be achieved by combining Airo navigation with navigated guide tubes for screw placement.

## 1. Introduction

Transforaminal lumbar interbody fusion (TLIF), as a modification of posterior lumbar interbody fusion (PLIF), was widely adopted to treat various lumbar disorders such as spondylolisthesis, stenosis, and instability. Due to its inherent far lateral approach to the spine, the incidence of the intraoperative durotomy and postoperative radiculitis was decreased compared with PLIF [1]. However, as a conventional open surgery, there are still concerns regarding more disruptive muscle detachment, extensive blood loss, long hospital stays, and more postoperative complications [2–5]. To overcome these flaws of open TLIF (O-TLIF), an alternative procedure of minimally invasive TLIF (MIS-TLIF) was developed and has become popular in the treatment of lumbar spinal disorders [6, 7]. MIS-TLIF is associated with minimal tissue disruption, due to its utilization of percutaneous or transfascial screw/rods, sequential dilation, and tubular retraction, which results in less tissue damage, reduced blood loss, lowered costs, and less hospitalization while maintaining similar clinical and radiographical outcomes compared to an O-TLIF [3, 5, 8–11]. However, some authors reported more radiation exposure and longer operative times in MIS-TLIF [11, 12]. According to two meta-analyses, the authors [5, 9] indicated no significant difference in operative time found between MIS-TLIF and O-TLIF. Yet, there was still significantly longer fluoroscopic exposure in the MIS-TLIF group compared with O-TLIF group. As computer-assisted surgery (CAS) has become more widely used in spine surgery, MIS-TLIF has become more accurate and safe, with a decrease in fluoroscopy exposure to the operating room (OR) staff and patient [13–15].

In MIS-TLIF, there is less direct visual access to the bony spine that increases the opportunity for surgical error. With the CAS and image-guided navigation, real-time and virtual intraoperative imaging of screws and their plotted trajectory is visible. This visibility greatly increases accuracy of instrumentation placement [16]. Recently, we used a portable intraoperative CT (iCT) scanner, Airo (Brainlab AG, Feldkirchen, Germany), combined with state-of-the-art computer navigation, not only for navigated instrumentation but also for intraoperative planning throughout the procedure, eliminating fluoroscopy. Therefore, the purpose of this study is to describe “total navigation” (i.e., complete intraoperative 3D navigation without fluoroscopy) with the Airo system used in all steps of MIS-TLIF.

## 2. Materials and Methods

### 2.1. Patients

**Figure 1:**
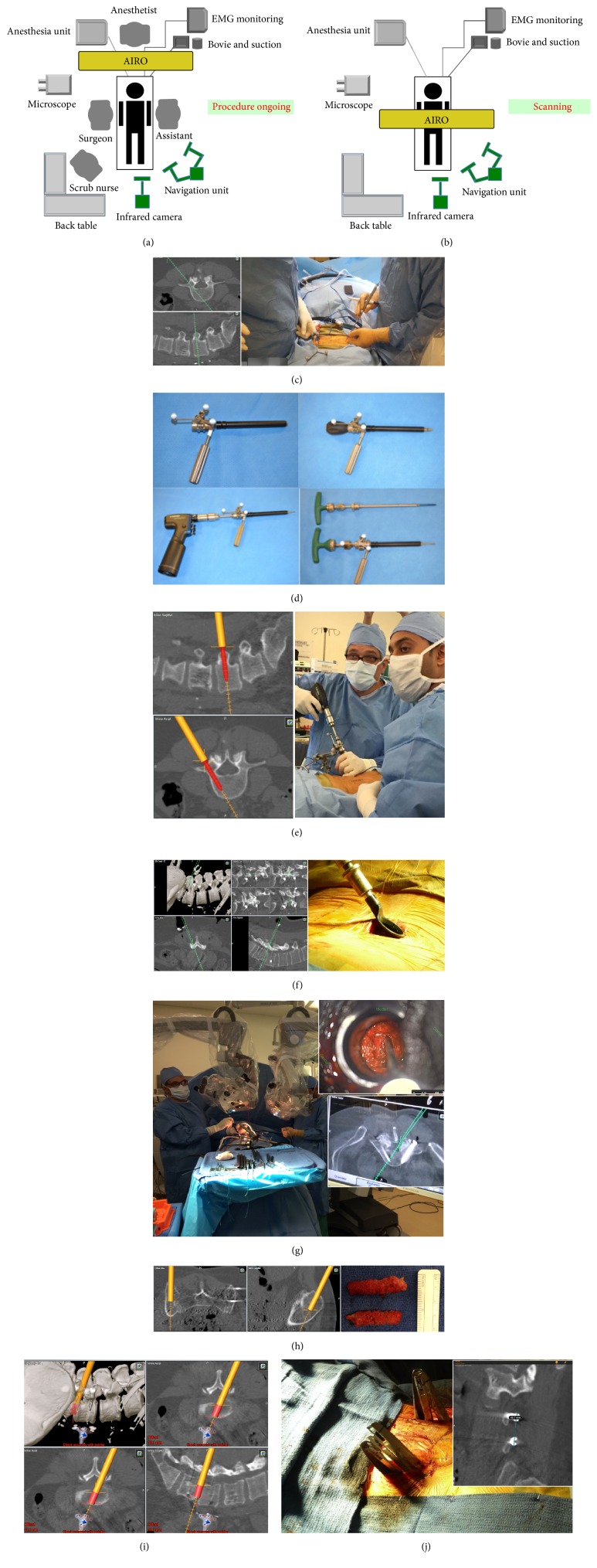
(a) iCT position during the procedure. (b) iCT position during active scan. (c) Skin incision planning. (d) The navigated guide tube allows dilating, drilling, tapping, and screw placement all through one navigated instrument. (e) Placement of pedicle screw. (f) Placement of tubular retractor with the insistence of navigation. (g) Determining extent of decompression by navigation. (h) Bone harvesting from iliac crest under guidance of navigation. (i) Cage planning. (j) Rod measurement from the screen of navigation station.

**Figure 2:**
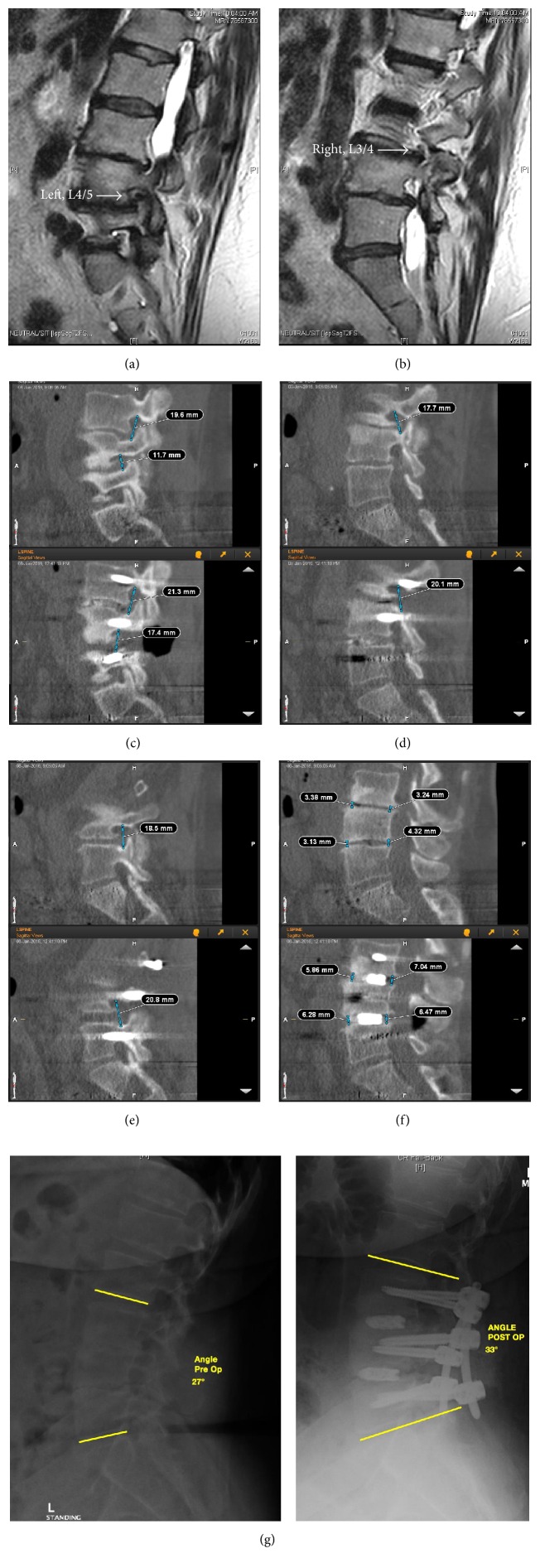
A 63-year-old female with two-level stenosis and instability. Pre-op MRI showed foraminal stenosis of left side in L4/5 (a) and right side in L3/4 (b). From navigation screen, foraminal heights were measured preoperatively and postoperatively. L3/4 and L4/5 of left side increased from 19.6 mm and 11.7 mm to 21.3 mm and 17.4 mm, respectively (c). L3/4 of right side increased from 17.7 mm to 20.1 mm (d). L4/5 of right side increased from 18.5 mm to 20.8 mm (e). The disc height of L3/4 and L4/5 increased postoperatively (f). The local lordosis improved postoperatively (g).

Between January 2015 and December 2015, a total of 33 consecutive patients were included in this study. The indications of these 33 patients included 9 patients (27.3%) with isthmic spondylolisthesis, 14 cases (42.4%) with degenerative spondylolisthesis, 3 cases (9.1%) with stenosis and instability, and 7 cases (21.2%) with spondylolisthesis secondary to postlaminectomy instability. In the 33 patients, there were 17 males and 16 females with a mean age of 66.6 years (range, 23–86). There were 27 cases with one level (Figure 1) and 6 cases with two levels (Figure 2) treated surgically. The segments of pathology were at L2/3 in one case, L3/4 in one case, L4/5 in 17 cases, L5/S1 in 8 cases, L3–5 in 1 case, and L4-S1 in 5 cases. All patients considered for surgery had low back pain and/or lower extremity pain and/or neurogenic intermittent claudication that were refractory to conservative treatment for no less than 3 months.

### 2.2. Workflow of Total Navigation with the Airo System

The setup of the iCT based Airo navigation system was shown in Figure 1. All patients underwent general anesthesia. After intubation, the patient is positioned prone to the radiolucent table which is perpendicular to the Airo CT scanner. All pertinent cables, such as the intubation tube, Bovie, and suction, are positioned with their leads going through the gantry of the Airo. The patient is taped to the table to ensure immobilization and increase accuracy of navigation.

The patient reference array is clipped onto the 2-pin fixator, which is attached to patient's pelvic using two 3 mm shank pins, and tightened into position. Two sterile half sheets are clipped around the incision and mark the scan range. To begin scanning, all personnel leave the OR, including the radiologic technologist, who brings the Airo touch screen outside the door to control the scan. Therefore, no lead apron is necessary for the surgeon or OR personnel. When the scan is complete, the images are automatically transferred to the Brainlab Curve*™* navigation system (BrainLab Curve, Brainlab AG, Feldkirchen, Germany).

The Brainlab Pointer is used to localize the pathology and identify the site of incision and its proper trajectory, which are then marked (Figure 1(c)). A navigated guide tube as previously described [17] is calibrated using the Brainlab Instrument Calibration Matrix (ICM4) and the Brainlab starlink array. The incision is made and the procedure begins, all while being navigated by the Brainlab Curve system. After skin incision has been made, accuracy is confirmed using the Brainlab Pointer by palpating a transverse process at a distance from the reference array. Then the screws are inserted through the navigated-tube guide (Figure 1(d)) bilaterally with transfascial style (Figure 1(e)).

Next, with the assistance of the pointer, the extent of bony decompression is determined and a tubular retractor is placed (Figure 1(f)), through which microsurgical decompression is conducted under microscopy (Figure 1(g)). Then, the disc is managed thoroughly. Bone graft is harvested from the iliac crest under the guidance of navigation (Figure 1(h)). The size and orientation of the cage are determined by the navigation (Figure 1(i)). After cage insertion, a second scan is made to verify the accuracy of all implants. Screw accuracy is classified into four grades at this stage according to Laine's criteria [18]. After the second scan, the appropriate length of the rod is measured using a pointer or from the screen (Figure 1(j)) and is then inserted. With full irrigation and hemostasis, the incisions are closed in routine style without drainage.

## 3. Results

All 33 patients were successfully operated on with the Airo navigation system without additional use of fluoroscopy. Although the first case utilized 5 scans, there were only six cases (18.2%) which utilized 3 scans, and the remaining 26 cases (78.8%) required only 2 scans. The average time of scans conducted by Airo was 2.3 in this series. The average effective radiation exposure of the patient of Airo was 5.47 mSv. There was no radiation exposure to the surgeon or OR personnel.

The Airo iCT scanner was moved into the OR when the patient was under intubation and anaesthetizing. No additional time was required for the setup of the navigation system. The average time from placement of reference array to first scan was 8.5 minutes (4–19 mins). Except for the first two cases, which took 19 and 18 minutes, respectively, all of them were less than 15 minutes and 24 cases (72.7%) were less than 10 minutes. The time from first scan to making first skin incision was 7.0 minutes (4–17 minutes), with the first two cases at 17 and 12 minutes, respectively. The remaining 31 cases (93.9%) were less than 10 minutes.

The average time of placement per screw was 2.6 minutes, almost half of them were placed by residents. The average time of the procedure was 192.8 minutes, with 175.6 minutes for a single level and 270.2 minutes for two levels.

For all patients, Airo navigation was utilized in pathology localization, bilateral incision planning, placement of tubular retractor, determination of the extent of bony decompression, determination of the size and orientation of cage, and measurement for proper rod length. In four cases, a tubular laminectomy in L1/2 was performed at the same time. In one case, an upper adjacent level tubular laminectomy and lower adjacent level foraminotomy were conducted. In these five cases, the other levels besides TLIF were localized and intraoperatively planned by navigation guidance.

A total of 144 screws were used in this series, with 4 screws per case in 27 cases and 6 screws per case in 6 cases. A postinstrumentation iCT scans showed 8 screws (5.6%) with minor perforations less than 2 mm and 2 screws (1.4%) misplaced with perforations >2–4 mm. All of the cortical breaches were lateral. No screws were misplaced > 4 mm. Using the Laine et al. [18] classification, the concluded effectiveness of Airo for accurate screw placement was 98.6%. There were no malpositioned screws in the verification scan. Intraoperative electromyography (EMG) monitoring was used for all patients when screws were being inserted. The average EMG output was 14.3 mA, all of them were between 9 mA and 20 mA except one screw was 4 mA. Intraoperative exploration confirmed that no breach was found in this screw.

No intraoperative complication was encountered in this series. All the scans were accurate as expected.

## 4. Discussion

From this study, we found that Airo navigation greatly facilitates MIS-TLIF throughout the procedure, not only for instrumentation with high rate accuracy, but also for intraoperative planning from pathology localization and skin incision to precise screw and cage placement, without any need for fluoroscopy. All the scans for target levels were accurate. The majority of the cases (78.8%) we performed using only two scans per case with the Airo, except for the first case in which 5 scans were done, reflecting the learning curve of this new technology.

Radiation exposure is one of the main hazards facing a surgeon during MIS procedures [19–21]. In a systematic review, Yu and Khan [22] reported that radiation exposure is greater to the surgeon, OR personnel, and patient in MISS procedures compared with open spine procedures. But utilization of advanced imaging with computer-assisted navigation in MIS procedures can decrease radiation exposure to the surgeon and OR personnel [22]. On the other hand, computer-assisted navigation based on intraoperative CT does result in increased radiation exposure to the patient compared with standard C-arm fluoroscopy; however, this is less than that with a standard diagnostic CT [23, 24]. In our series, there is no radiation exposure to the surgeon and OR staff, no lead aprons required, and therefore less possibility for lower back pain in the surgeon. For patients, if the postoperative CT scans were considered to check the accuracy of instrumentation, the radiation exposure would be greater when compared with Airo iCT.

One concern is that more time is required for setup of the navigation system. In a cadaver study, Tabaraee et al. [25] demonstrated that compared to conventional fluoroscopy, setup of O-arm iCT navigation system needs more time, but placement of pedicle screws requires less time. The overall time of these two techniques was similar. Yang et al. [26] demonstrated an average time of 3.65 minutes for guide-wire placement per pedicle using fluoroscopy-based navigation to place percutaneous pedicle screws in the lumbar spine. In our study, instead of using K-wires for screw placement, we use a navigated-tube guide as described previously [17] to insert the pedicle screw. This greatly increased the workflow due to drill, tapping, and screw insertion via the same tube. This may be attributed to less time required for placing individual screws in our series, which took 2.6 minutes on average, compared to previous studies [25–27]. Although there is more time required to set up the Airo iCT navigation, the total time for the whole procedure is comparable. However, further comparison studies should be performed.

Due to less direct visualization of anatomical landmarks, the opportunity for malposition or perforation of instrumentation would increase in MISS. Without navigation, pedicle perforation after percutaneous pedicle screw insertion was reported to be more than 10% [28, 29]. A meta-analysis showed overall perforation risk of 13.1% when 2D fluoroscopic navigation was utilized in MISS [30]. With Airo 3D image-guided navigation, real-time, virtual, intraoperative imaging of screws and their plotted trajectory is possible. This enhanced visibility enables the surgeon to place the required instrumentation more accurately, with a rate of accuracy at 98.6% in current study. To increase the accuracy, we have developed some tips including taping the patient to OR table using battery driven drill to avoid pressure to the patient and constant comparison of the tactile feedback with the navigation screen to confirm accuracy of the system. Before, during, and after any crucial step like intervertebral disc removal or pedicle screw or drill insertion we match calibration of the probes with anatomical landmarks like the transvers process; if any anomaly is detected the patient has to be draped and a second scan must be performed before proceeding. Also, transoperative X-ray intensifier can be used while the intervertebral cages are inserted.

From present study, we found that Airo based navigation greatly facilitates MIS-TLIF throughout the procedure without any need for fluoroscopy. Thus, an era of “total navigation” was introduced in MISS, that is, the use of navigation for all steps of the procedure, from pathology localization, incision planning to screw placement, tubular decompression, cage placement, and rod measurement and without the need for fluoroscopy. However, this requires slight modification of the nonnavigated MIS technique in order to maximize navigation accuracy.

The limitation of current study is the lack of a matched controlled group and its small number of patients. In the future, a prospective randomized controlled study with a larger population should be performed to determine the extra benefits of the total navigation. Also, it should be addressed in future studies whether more accurate instrumentation with navigation correlates with better clinical outcomes.

## 5. Conclusion

The Airo based IGS greatly expands navigation from a tool used solely for instrumentation navigation to one used for intraoperative planning throughout the whole procedure in MIS-TLIF. It has introduced an era of “total navigation,” that is, the use of navigation for all steps of the procedure, from pathology localization, incision planning to screw placement, tubular decompression, cage placement, and rod measurement and without the need for fluoroscopy. This present study shows that navigation provides a high rate of screw accuracy without adding additional time to the surgery.
